# California and federal school nutrition policies and obesity among children of Pacific Islander, American Indian/Alaska Native, and Filipino origins: Interrupted time series analysis

**DOI:** 10.1371/journal.pmed.1003596

**Published:** 2021-05-24

**Authors:** Mika Matsuzaki, Brisa N. Sánchez, R. David Rebanal, Joel Gittelsohn, Emma V. Sanchez-Vaznaugh

**Affiliations:** 1 Johns Hopkins Bloomberg School of Public Health, Department of International Health, Center for Human Nutrition, Baltimore, Maryland, United States of America; 2 Drexel University Dornsife School of Public Health, Department of Biostatistics, Philadelphia, Pennsylvania, United States of America; 3 San Francisco State University, Health Equity Institute, San Francisco, California, United States of America; 4 San Francisco State University, Department of Public Health, San Francisco, California, United States of America; 5 University of California San Francisco, Center for Health Equity, San Francisco, California, United States of America; Carolina Population Center, UNITED STATES

## Abstract

**Background:**

Obesity prevalence remains high among children of Pacific Islander (PI) origin, Filipino (FI), and American Indian/Alaska Native (AIAN) origins in the United States. While school nutrition policies may help prevent and reduce childhood obesity, their influences specifically among PI, FI, and AIAN children remain understudied. We evaluated the association of the California (CA) state school nutrition policies for competitive food and beverages and the federal policy for school meals (Healthy, Hunger-Free Kids Act of 2010 (HHFKA 2010)) with overweight/obesity among PI, FI, and AIAN students.

**Methods and findings:**

We used an interrupted time series (ITS) design with FitnessGram data from 2002 to 2016 for PI (78,841), FI (328,667), AIAN (97,129), and White (3,309,982) students in fifth and seventh grades who attended CA public schools. Multilevel logistic regression models estimated the associations of the CA school nutrition policies (in effect beginning in academic year 2004 to 2005) and HHFKA 2010 (from academic year 2012 to 2013) with overweight/obesity prevalence (above the 85 percentile of the age- and sex-specific body mass index (BMI) distribution). The models were constructed separately for each grade and sex combination and adjusted for school district-, school-, and student-level characteristics such as percentage of students eligible for free and reduced price meals, neighborhood income and education levels, and age. Across the study period, the crude prevalence of overweight/obesity was higher among PI (39.5% to 52.5%), FI (32.9% to 36.7%), and AIAN (37.7% to 45.6%) children, compared to White (26.8% to 30.2%) students. The results generally showed favorable association of the CA nutrition policies with overweight/obesity prevalence trends, although the magnitudes of associations and strengths of evidence varied among racial/ethnic subgroups. Before the CA policies went into effect (2002 to 2004), overweight/obesity prevalence increased for White, PI, and AIAN students in both grades and sex groups as well as FI girls in seventh grade. After the CA policies took place (2005 to 2012), the overweight/obesity rates decreased for almost all subgroups who experienced increasing trends before the policies, with the largest decrease seen among PI girls in fifth grade (before: log odds ratio = 0.149 (95% CI 0.108 to 0.189; *p* < 0.001); after: 0.010 (−0.005 to 0.025; 0.178)). When both the CA nutrition policies and HHFKA 2010 were in effect (2013 to 2016), declines in the overweight/obesity prevalence were seen among White girls and FI boys in fifth grade. Despite the evidence of the favorable association of the school nutrition policies with overweight/obesity prevalence trends, disparities between PI and AIAN students and their White peers remained large after the policies took place. As these policies went into effect for all public schools in CA, without a clear comparison group, we cannot conclude that the changes in prevalence trends were solely attributable to these policies.

**Conclusions:**

The current study found evidence of favorable associations of the state and federal school nutrition policies with overweight/obesity prevalence trends. However, the prevalence of overweight/obesity continued to be high among PI and AIAN students and FI boys. There remain wide racial/ethnic disparities between these racial/ethnic minority subgroups and their White peers. Additional strategies are needed to reduce childhood obesity and related disparities among these understudied racial/ethnic populations.

## Abstract 2

Dr. Mika Matsuzaki and co-authors found evidence of favorable associations between state and federal school nutrition policies with overweight/obesity prevalence trends among children of Pacific Islander origin, Filipino, and American Indian/Alaska Native origins in the United States.

## Author summary

**Why was this study done?:**

- Governmental school nutrition policies can potentially have a wide-reaching impact on children’s dietary behaviors and health.
- It is currently unclear whether state and national school nutrition policies are equally effective among children of different racial/ethnic backgrounds.
- Children of Pacific Islander (PI), Filipino (FI), and American Indian/Alaska Native (AIAN) origins are some of the most understudied subgroups experiencing high prevalence of overweight/obesity.

**What did the researchers do and find?:**

- We used an interrupted time series (ITS) design to examine whether state and national school policies in the United States were associated with favorable changes in overweight/obesity prevalence among PI, FI, and AIAN children.
- During the baseline period without any policies, the overweight/obesity prevalence increased among nearly all subgroups, with steeper increases for PI and AIAN students.
- After the California (CA) competitive foods and beverages (CF&B) policy took place, the rates of change in overweight/obesity prevalence decreased for most subgroups.
- There was weaker evidence for favorable changes in overweight/obesity prevalence after the addition of the national policy.
- Decreasing trends in overweight/obesity prevalence following the school nutrition policies were seen for some of the White and FI students but not for PI or AIAN students.

**What do these findings mean?:**

- The study found some evidence of favorable changes in overweight/obesity prevalence following the state and national school nutrition policies.
- Regulation on CF&B may be an especially effective strategy to reduce overweight/obesity among schoolchildren.
- There remain large disparities between PI and AIAN children and FI boys and their White peers.
- There is a need to further strengthen policies to improve food environments for children from racial/ethnic minority populations with high obesity prevalence.

## Introduction

Obesity during youth is associated with short- and long-term health consequences such as diabetes, cancer, hypertension, and atherosclerosis [1,2]. In 2015 to 2016, the overall national estimate of childhood obesity in the United States was 18.4% and over 20% among adolescents between ages of 12 and 19 years [3]. The prevalence of obesity in children and adolescents varies among racial/ethnic subgroups; however, data on children of Pacific Islander (PI) origin and Asian backgrounds are relatively scarce [3–5]. Reports have generally shown lower prevalence of obesity among Asian youth as a whole in comparison to other racial/ethnic populations. However, Asians are a heterogeneous group constituting racially/ethnically diverse subgroups with varying characteristics including obesity. Children of Filipino (FI) origin and Southeast Asian backgrounds have higher overweight/obesity prevalence (29.5% and 27.3%, respectively) than those of Chinese, Japanese, Korean, and South Asian racial/ethnic origins [6]. American Indian/Alaska Native (AIAN) children had a comparably high prevalence at 29.7% in 2015 [7]. While studies and reports suggested high prevalence of obesity among PI youth, this subgroup is often classified into the Asian racial/ethnic group [8] or excluded from analyses, leading to limited availability of data on obesity prevalence and related chronic diseases among PIs [9,10].

Over the past 2 decades, many strategies have been adopted to change the upward trajectories in childhood obesity through policies targeting the school food environment [11–14]. As of October 1, 2010, 39 states had enacted policies for “competitive” foods in schools—foods whose availability “compete” with federally reimbursable meals [11]. A previous study reported that most commonly consumed competitive foods and beverages (CF&B) are energy dense and nutrient poor [15]. California (CA) was among the first states to enact CF&B policies [16], starting in 2002, with the aim to reduce the rising burden of childhood obesity in the state. These policies prohibited the sale of beverages with added sugar and regulate nutrient content of snacks in elementary schools and snacks and entrees in middle schools. In 2007, CA further banned fried foods and *trans*-fat in school meals [17,18]. In addition to CA’s CF&B policies, the Healthy, Hunger-Free Kids Act of 2010 (HHFKA 2010) was enacted in 2010 as a federal policy to improve nutrition standards in federally reimbursed school meals, which went into effect in the academic year of 2012 to 2013. To our knowledge, no other study has examined the associations between these school nutrition policies and childhood obesity specifically among PI, AIAN, and FI students.

The paucity of research on nutrition policy influences on obesity among PI, AIAN, and FI students is in part due to their insufficient sample sizes in national surveys and higher concentrations in certain geographic locations. Because CA is home to some of the largest populations of people with FI and PI origins as well as AIs in the US [19–21], it is an ideal state to study potential influences of school nutrition policies among these understudied subgroups. Using demographic, body composition, and fitness data from the California Department of Education (CDE) [22], the current study aimed to evaluate the associations of CA and federal school nutrition policies with obesity prevalence among PI, AIAN, and FI students. Because many racial/ethnic subgroups are more likely to be eligible for and thus participate in school meals than their White peers, the school nutrition policies carried a potential to reduce obesity prevalence for these subgroups and to contribute to the mitigation of the existing obesity disparities. The findings provide among the first insights into whether state and federal regulation of school meals, food, snacks, and beverages may have had variable levels of association with overweight/obesity rates in these understudied racial/ethnic subgroups.

## Materials and methods

This study is reported as per the Strengthening the Reporting of Observational Studies in Epidemiology (STROBE) guidelines (S1 Checklist).

### Settings

The current study used repeated cross-sectional data from public schools in the state of CA in the US, which were required to implement the school nutrition policies.

### Participants

The study used data from the students who participated in the Physical Fitness Testing in public schools in CA who (1) were in fifth or seventh grade each year between 2002 and 2016; (2) reported racial/ethnic groups of PI, AIAN, FI, or White; (3) did not have any missing data on covariates and outcome variables; and (4) attended schools that were required by law to implement the new nutrition standards.

### Data sources/measurement

Height and weight information was measured by school staff as part of the Physical Fitness Testing, administered annually to public school students in fifth, seventh, and ninth grade [22]. These data have been shown to be valid and reliable, in comparison to data collected by trained specialists [23]. The database—FitnessGram—includes information on age, sex, race/ethnicity, and grade, in addition to levels of physical fitness. Yearly child-level FitnessGram data between 2002 and 2016 were merged with information from 3 additional sources: unique and geocoded school addresses publicly available through CDE; 2000 and 2010 Censuses; and the 2015 American Community Survey.

### Variables

#### Exposure

The exposure variables were the years in which CA state and federal school nutrition policies went into effect. Since 2004, CA has regulated the nutritional content and availability of beverages in elementary and middle schools. In 2007, CA introduced statewide nutrition and portion size standards for competitive foods in grades K-12. The policies set the maximum number of calories in snacks or entrees and percent of calories from fat at ≤35% and from saturated fat to ≤10% and sugar content to no more than 35% by weight. In the same year, CA also banned fried foods and *trans*-fats in school meals. The federal policy—HHFKA 2010—sought to improve nutrition standards for school meals as follows: set caloric limits, and increased daily availability of fruits, vegetables, and whole grain–rich foods, limited fat content of milk and portion sizes, and reduced levels of sodium, saturated fat, and *trans*-fat [24]. HHFKA 2010 also set nationwide standards for CF&B effective in the academic year of 2014 to 2015, which were mostly similar to those already in place for nearly 10 years in CA. Details of the CA and federal nutrition policies can be found elsewhere [24,25].

#### Outcomes

Overweight/obesity status was the primary outcome. Body mass index (BMI) was calculated (weight in kilograms (kg) per height in meters squared (m^2^)), and BMI z-scores for each age and sex were derived. Students were categorized as normal/underweight or overweight/obese (above 85 percentile for BMI-z) using the growth chart in 2000 from the Center for Disease Control [26].

### Potential confounders and effect modifiers

#### Student-level data

We included age in years (as of April 1 of the year in which students took the FitnessGram test), sex, racial/ethnic origins/backgrounds (PI, AIAN, FI, and White), and fitness levels based on whether students exceeded, met, or did not meet the Cooper Institute’s guidelines for the time to run a mile for each age and sex. If students had missing mile-run data, the Progressive Aerobic Cardiovascular Endurance Run (PACER) data were used instead by converting the number of laps in the PACER test into minutes needed to run a mile using a published equivalency chart.

#### School-level data

The average total number of enrolled students within each school each year was calculated by using all available yearly data between 2002 and 2016. The percentage of White students for each school was calculated for each year. Student-level socioeconomic data were not available, and therefore, the proportions of students eligible for free or reduced price meals (FRPM) for each school for each year was used as a proxy of children’s socioeconomic characteristics. We also used 2 additional school neighborhood socioeconomic indicators: the median household income and proportions of the residents achieving a bachelor’s degree or above within each census tract where schools were located. The values for each school’s census track were used to interpolate a value of the socioeconomic indicators for each year.

#### School district data

District-level FRPM variable was constructed by aggregating school-level data to the district level and splitting into quartiles.

### Statistical analysis

The analyses were initially planned in October 2016 as part of a grant submission and refined in October 2020, in response to the peer review process as well as updated information about schools that the legislation required to implement the CA nutrition policies.

#### Descriptive analyses

Student-level (age, grade, sex, fitness level, and overweight and obesity prevalence) and school-level characteristics (educational attainment, median household income, average number of total enrolled students, and % eligible for FRPM) are presented for each racial/ethnic subgroup (PI, AIAN, FI, and White). For the school-level characteristics, the median values for each racial/ethnic subgroup are presented.

#### Modeling strategy

The prospectively specified modeling strategy is as follows. Nested 3-level logistic regression models were fit to estimate the associations of the CA and federal nutrition policies with overweight/obesity, while accounting for student-level characteristics and school- and school district–level clustering. The fixed effects in the model followed an interrupted time series (ITS) design specification, wherein calendar year was used to model the population level overweight/obesity trend and 2 linear splines, with knots at 2005 (for the CA policies) and 2013 (for the federal policies) were used to model the trend changes concomitant with the policies after they went into effect. That is, the policies interrupt the population-level upward trend in overweight/obesity that would otherwise be observed had the policies not occurred. The knots were chosen as 2005 and 2013 because they correspond to the spring in the first academic year when the policies went into effect. The ITS analysis assumes that changes in overweight/obesity prevalence were gradual. Thus, the model only included a change in the slope in the population-level trend in overweight/obesity associated with the policies, but not a discontinuous change in the level or prevalence in overweight/obesity. A change in the overweight/obesity levels concomitant with the start dates of the policies would assume the policies has an immediate effect on body weight, which is unlikely because body weight changes are expected to occur gradually overtime.

Terms for race/ethnicity as well as the interaction of race/ethnicity with terms for calendar time and splines were included to examine racial/ethnic differences in the population-level overweight/obesity prevalence and trend. Random effects were used for the intercept and each of the calendar year terms and were assumed to have a multivariate normal distribution with unstructured covariance matrix at both school and school district levels. The models were adjusted for the student-level covariates, and school and school neighborhood time-varying confounders described above. Based on these models, we compared estimated yearly changes (“slopes”) in overweight/obesity prevalence across 3 periods: before either the CA or federal policy took place (2002 to 2004), CA policy only period (2005 to 2012), and after the addition of the federal policy (2013 to 2016). We compared the trend in overweight/obesity from each period to the period before, as well as the cumulative change from the period without any policy to the period when both policies were in effect (2002 to 2004 versus 2013 to 2016). We also compared differences in these yearly changes between each of the racial/ethnic subgroups and White students to understand changes in overweight/obesity disparities. Formal tests of differences in the annual log odds of obesity comparing after to before each policy were conducted (S1–S4 Tables). All models were stratified by grade and sex. Detailed description of the models are included in S1 Method.

Model results were also used to estimate annual prevalence. We compared the prevalence estimates from the model that assumes the policies are in place with “counterfactual prevalence” that could have been observed had the pre-policy trend in overweight/obesity continued. These counterfactual estimates were obtained by linearly projecting the trends in the log odds of overweight/obesity from the pre-policy (2002 to 2004) through 2005 to 2016. We then calculated the difference between the estimated prevalence with the policies in place versus the counterfactual prevalence. The same models were constructed with obesity as the outcome also. Analyses were conducted in the R statistical package. The models were fitted using quasi-likelihood via the glmmPQL function. Unless otherwise stated, all *p*-values shown in the results are derived from tests of whether a regression coefficient or a combination of regression coefficients is different from 0.

As the data for this study were provided by the CDE, it was reviewed by and received approval from the Committee for the Protection of Human Subjects of the CA’s Office of Statewide Health Planning and Development. However, as a secondary data analysis study, it was exempt from review by the author’s institutions.

### Limitations

Although the ITS design enables robust inferences about the potential effects of the policies without the need for a control group [27], the study cannot definitively conclude the causal relationships because it did not have clear comparison groups who were unexposed to the state and federal policies as these policies went into effect at all public schools. The analysis uses data on children with complete information. Given that the regression model adjusts for covariates that are also predictors of missing data, the inferences derived from them are likely to have minimal bias assuming the missing data are missing at random [28].

## Results

Of the 12,620,300 child-level observations for fifth and seventh graders in the FitnessGram data set between 2002 and 2016, 94,540 (0.7%), 118,261 (0.8%), and 371,708 (2.7%) pertained to PI, AIAN, and FI students and 3,855,298 pertained to White children. Among these, >94.4% in each subgroup attended schools where the policies were known to apply, and thus were eligible for this study (*n* = 90,098 PI; 111,630 AIAN; 361,209 FI; and 3,685,340 White). Among the eligible, 78,841 (87.5%) PI, 97,129 (87.0%) AIAN, 328,667 (91.0%) FI, and 3,309,982 (89.8%) White students had complete data and were included in this study (Table 1). Among the included children, comparable percentages of FI and White students met or exceeded the fitness standard (68% and 74%, respectively) while smaller proportions of PI and AIAN students achieved the same (63% and 62%).

**Table 1:** Characteristics of students by race/ethnicity^1^.

Characteristics	PI Total^2^	*n* (%)	AIAN Total	*n* (%)	FI Total	*n* (%)	White Total	*n* (%)
Grade								
Fifth	78,841	40,037 (50.8)	97,129	49,831 (51.3)	328,667	165,646 (50.4)	3,309,982	1,673,741 (50.6)
Seventh	78,841	38,804 (49.2)	97,129	47,298 (48.7)	328,667	163,021 (49.6)	3,309,982	1,636,241 (49.4)
Sex								
Boys	78,841	40,125 (50.9)	97,129	48,905 (50.4)	328,667	171,001 (52.0)	3,309,982	1,703,654 (51.5)
Girls	78,841	38,716 (49.1)	97,129	48,224 (49.7)	328,667	157,666 (48.0)	3,309,982	1,606,328 (48.5)
Age								
9	78,841	36 (0.05)	97,129	26 (0.03)	328,667	114 (0.03)	3,309,982	862 (0.03)
10	78,841	22,144 (28.1)	97,129	25,176 (25.9)	328,667	94,910 (28.9)	3,309,982	863,439 (26.1)
11	78,841	16,907 (21.4)	97,129	22,831 (23.5)	328,667	67,854 (20.7)	3,309,982	782,045 (23.6)
12	78,841	22,667 (28.8)	97,129	25,782 (26.5)	328,667	94,482 (28.8)	3,309,982	876,234 (26.5)
13	78,841	16,049 (20.4)	97,129	21,539 (22.2)	328,667	67,753 (20.6)	3,309,982	757,007 (22.9)
14	78,841	1,025 (1.3)	97,129	1,745 (1.8)	328,667	3,486 (1.1)	3,309,982	29,718 (0.9)
15	78,841	13 (0.02)	97,129	30 (0.03)	328,667	68 (0.02)	3,309,982	677 (0.02)
Fitness level^3^								
Unfit	78,841	29,040 (36.8)	97,129	37,217 (38.3)	328,667	104,514 (31.8)	3,309,982	870,070 (26.3)
Fit	78,841	36,557 (46.4)	97,129	42,903 (44.2)	328,667	166,394 (50.6)	3,309,982	1,544,151 (46.7)
Super fit	78,841	13,244 (16.8)	97,129	17,009 (17.5)	328,667	57,759 (17.6)	3,309,982	895,761 (27.1)
Overweight/obesity^4^								
2001–2002	6,859	2,797 (40.8)	6,939	2,614 (37.7)	18,808	6,607 (35.1)	228,723	66,929 (29.3)
2002–2003	8,451	3,334 (39.5)	7,633	3,083 (40.4)	19,726	7,170 (36.4)	250,803	74,634 (29.8)
2003–2004	5,759	2,679 (46.5)	6,488	2,681 (41.3)	20,067	7,124 (35.5)	247,642	73,586 (29.7)
2004–2005	5,764	2,723 (47.2)	6,156	2,632 (42.8)	21,880	8,035 (36.7)	241,538	73,038 (30.2)
2005–2006	5,087	2,589 (50.9)	5,923	2,529 (42.7)	22,398	7,956 (35.5)	232,758	68,584 (29.5)
2006–2007	4,935	2,539 (51.5)	5,970	2,617 (43.8)	22,630	7,927 (35.0)	227,694	65,946 (29.0)
2007–2008	5,174	2,685 (51.9)	6,163	2,616 (42.5)	23,951	8,454 (35.3)	226,316	65,669 (29.0)
2008–2009	5,326	2,740 (51.5)	6,096	2,678 (43.9)	24,578	8,685 (35.3)	224,730	64,904 (28.9)
2009–2010	5,215	2,668 (51.2)	6,774	3,022 (44.6)	21,429	7,583 (35.4)	213,321	62,011 (29.1)
2010–2011	4,663	2,439 (52.3)	5,055	2,222 (44.0)	22,436	7,885 (35.1)	202,028	56,495 (28.0)
2011–2012	4,587	2,294 (50.0)	5,352	2,382 (44.5)	23,292	7,930 (34.1)	209,033	58,092 (27.8)
2012–2013	4,408	2,247 (51.0)	5,193	2,271 (43.7)	22,826	7,672 (33.6)	206,923	56,050 (27.1)
2013–2014	4,180	2,057 (49.2)	4,678	2,062 (44.1)	21,755	7,265 (33.4)	200,422	53,773 (26.8)
2014–2015	4,260	2,238 (52.5)	9,624	4,385 (45.6)	21,644	7,118 (32.9)	199,838	53,587 (26.8)
2015–2016	4,173	2,086 (50.0)	9,085	4,146 (45.6)	21,247	6,991 (32.9)	198,213	53,803 (27.1)

^1^ Authors’ analysis of the CA FitnessGram data. The numbers are *n* (% within each racial/ethnic group) unless otherwise noted.

^2^ Total *n* for overweight/obesity is for each academic year for each racial/ethnic group. For all other characteristics, the total *n* is for each racial/ethnicity.

^3^ Those categories are based on whether students exceeded, met, or did not meet the Cooper Institute’s guidelines for the time to run a mile for each age and sex.

^4^ Overweight and obese categories are defined as having BMI z-score 85 and 95 percentiles in comparison to the Centers for Disease Control and Prevention’s growth chart from 2000.

AIAN, American Indian/Alaska Native; BMI, body mass index; CA, California; FI, children of Filipino origin; PI, children of Pacific Islander origin.

**Fig 1:**
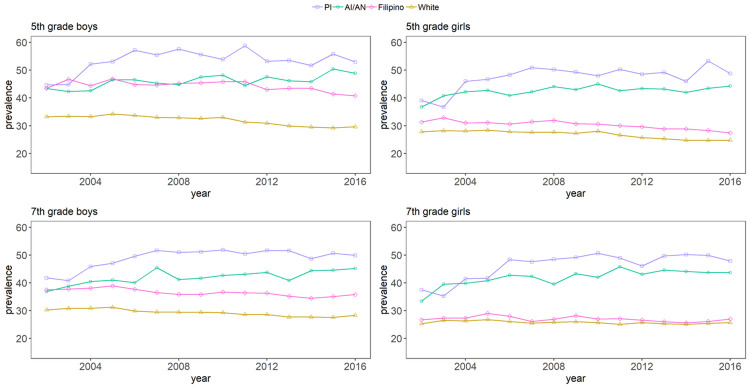
Observed overweight/obesity prevalence by grade, sex, and race/ethnicity. AI/AN, American Indian/Alaska Native; PI, Pacific Islander.

The crude prevalence of overweight/obesity was higher among PI, AIAN, and FI students across all study years when compared to White students (Table 1). The crude prevalence initially increased sharply for PI and AIAN students and stabilized thereafter (Table 1). The crude overweight/obesity prevalence stratified by grade and sex revealed several distinct patterns (Fig 1). The prevalence was higher among fifth graders than seventh graders, and across all racial/ethnic subgroups, boys generally had higher prevalence than girls. The sex difference was most profound among FI students, where the prevalence for girls was low and closer to their White peers than the other racial/ethnic and sex subgroups.

Schools attended by PI, AIAN, and FI students had higher median values for the proportions of students eligible for FRPM than those attended by White students. Neighborhoods of schools attended by PI and AIAN students had lower values for the proportions of neighborhood residents completing a college degree and median annual household income levels than FI or White students (Table 2).

**Table 2:** Characteristics of schools by racial/ethnic subgroups.

Characteristics	PI	AIAN	FI	White
Free and reduced meal eligible students^1^ (%)				
2002	51.1	45.2	42.3	27.6
2005	49.6	49.9	46.3	28.8
2012	64.6	61.8	50.6	35.5
School neighborhood education level^2^ (%)				
2002	23.4	19.3	25.3	27.7
2005	23.6	19.6	26.0	29.5
2012	25.2	21.6	31.7	34.4
School neighborhood median income (US dollars)				
2002	49,909	49,432	57,717	59,554
2005	59,908	51,009	63,442	65,657
2012	65,610	56,818	76,824	77,494
Median school enrollment^3^ (*n*)				
2002	811	657	754	700
2005	745	670	782	713
2012	748	641	776	704

Median values are shown for each subgroup unless otherwise noted.

^1^ Authors’ analysis of school characteristics databases, available publicly within the CDE’s website.

^2^ Defined as percentage of residents who completed a bachelor’s degree or more.

^3^ Mean of yearly enrollment data across all years for each school.

To understand the relationships between these policies and overweight/obesity, we examined the adjusted (1) prevalence trends in 2002–2016; (2) changes in overweight/obesity prevalence after each policy introduction; and (3) differences in changes in prevalence between each minority subgroup and White students after the introduction of each policy. The numerical values of the estimated associations and comparisons between periods are included in S1–S4 Tables.

AIAN, American Indian/Alaska Native; CDE, California Department of Education; FI, children of Filipino origin; PI, children of Pacific Islander origin.

**Fig 2:**
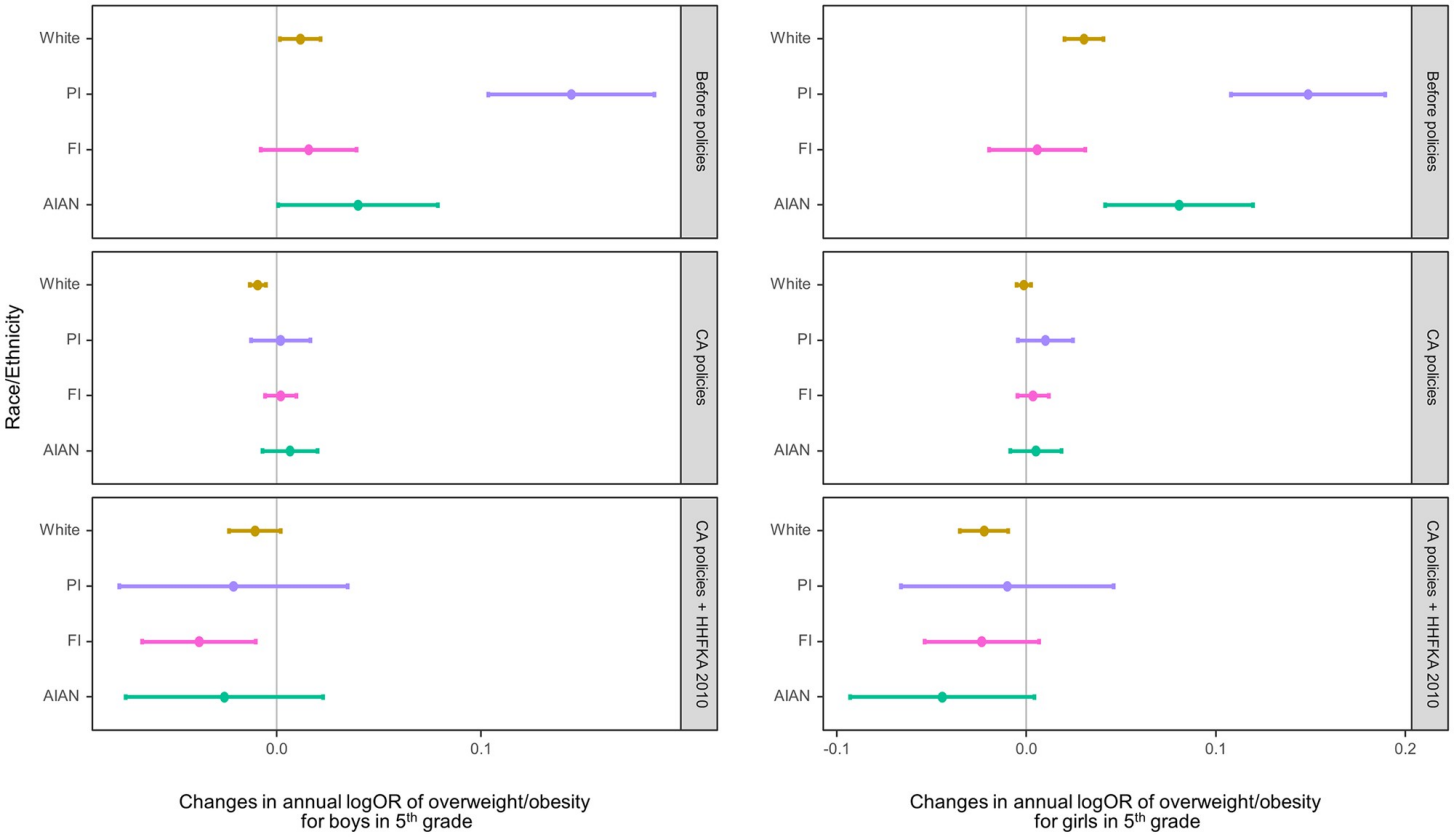
Adjusted change in the log odds of overweight/obesity per year within the periods 2002–2004 (baseline, no policies in effect), 2005–2012 (after CA policies and before HHFKA 2010), and 2013–2016 (CA policies plus HHFKA 2010) among fifth graders by sex. The dots indicate the point estimates of the yearly changes in the log odds, and the whiskers show the 95% confidence intervals. AIAN, American Indian/Alaska Native; CA, California; FI, children of Filipino origin; HHFKA 2010, Healthy, Hunger-Free Kids Act of 2010; OR, odds ratio; PI, children of Pacific Islander origin; W, White.

**Fig 3:**
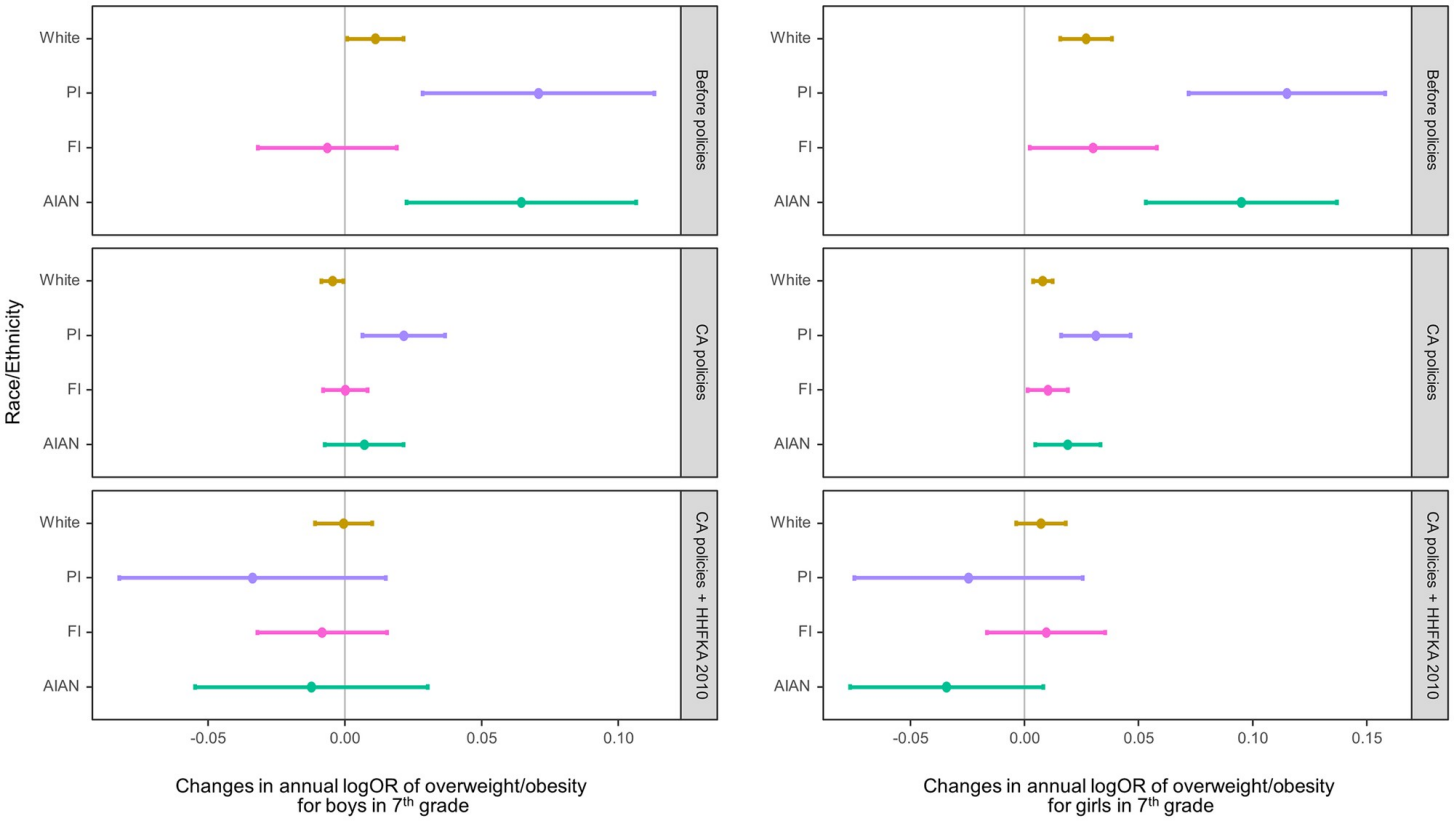
Adjusted change in the log odds of overweight/obesity per year within the periods 2002–2004 (baseline, no policies in effect), 2005–2012 (after CA policies and before HHFKA 2010), and 2013–2016 (CA policies plus HHFKA 2010) among seventh graders by sex. The dots indicate the point estimates of the yearly changes in the log odds, and the whiskers show the 95% confidence intervals. AIAN, American Indian/Alaska Native; CA, California; FI, children of Filipino origin; HHFKA 2010, Healthy, Hunger-Free Kids Act of 2010; OR, odds ratio; PI, children of Pacific Islander origin; W, White.

Figs 2 and 3 show the estimated yearly changes in log odds of overweight/obesity among fifth and seventh graders, respectively, by race/ethnicity within each of the 3 periods: baseline (2002 to 2004), after CA CF&B policy before the federal policy (2005 to 2012), and after the federal policy (i.e., when both CA and federal policies were in place; 2013 to 2016). In the figures, positive values indicate an increasing trend in overweight/obesity prevalence; negative values indicate a decreasing trend, while values that are not different from 0 represent a plateau. During the baseline period without any policies, the overweight/obesity prevalence was increasing among almost all subgroups—with the exception of FI fifth graders and FI boys in seventh grade—with PI and AIAN students having the steeper increases.

After the CA CF&B policy took place, the rates of increases in overweight/obesity prevalence decreased for all White students, PI and AIAN girls in both grades, and PI boys in fifth grade and AIAN boys in seventh grade (Figs 2 and 3, S1–S4 Tables). There was no strong evidence of changes in the overweight/obesity rates for FI students in either grade, AIAN boys in fifth grade, and PI boys in seventh grade after the CA CF&B policy went into effect. The changes in rates following the state policies led to no substantial changes in overweight/obesity prevalence in 2005 to 2012 for all 4 subgroups in fifth grade, except for White boys whose prevalence decreased (adjusted logOR = −0.009 (95% CI: −0.013 to −0.005; *p* < 0.001). The directions of prevalence trends during this period were more variable for seventh graders. Among seventh graders, the prevalence continued to increase for all girls and PI boys, although at a slower rate than the period before. The prevalence plateaued for AIAN and FI boys (adjusted logOR = 0.007 (95% CI: −0.007 to 0.021; *p* = 0.337) and 0.000 (−0.008 to 0.008; 0.971), respectively). Similar to White boys in fifth grade, there was evidence of a decreasing trend among White boys in seventh grade, although the magnitude was small (adjusted logOR = −0.005 (95% CI: −0.009 to −0.001; *p* = 0.025).

After HHFKA 2010 for school meals went into effect, most subgroups showed no substantial changes in the overweight/obesity rates from the period before, except for White girls and FI boys in fifth grade, whose overweight/obesity prevalence rates decreased in comparison to the period before. During this period when both state and federal policies were in effect (2013 to 2016), decreasing trends in overweight/obesity were seen only for White girls and FI boys in fifth grade; for all other subgroups, the prevalence remained stable.

When comparing the prevalence trends during the baseline period (2002 to 2004) to those in the period when both CA and federal policies were in effect (“cumulative association”), there was evidence of favorable changes in the prevalence trends for all groups except for FI girls in both grades and White and FI boys in seventh grade. In other words, after both of the policies were in place, our models estimated that the trajectories of overweight/obesity changed to either a plateau or a decreasing trend for most subgroups. The same models with obesity as the outcome, instead of overweight/obesity, did not materially change the results.

If we were to assume unchanged, linear overweight/obesity trajectories in a scenario where no policies were put in place, the 2016 overweight/obesity prevalence could have been 11% (AIAN) and 32% (PI) among boys and 24% (AIAN) and 34% (PI) points higher among girls in fifth grade and 17% (AIAN, PI) among boys and 24% (AIAN) and 25% (PI) points higher among girls in seventh grade.

**Fig 4:**
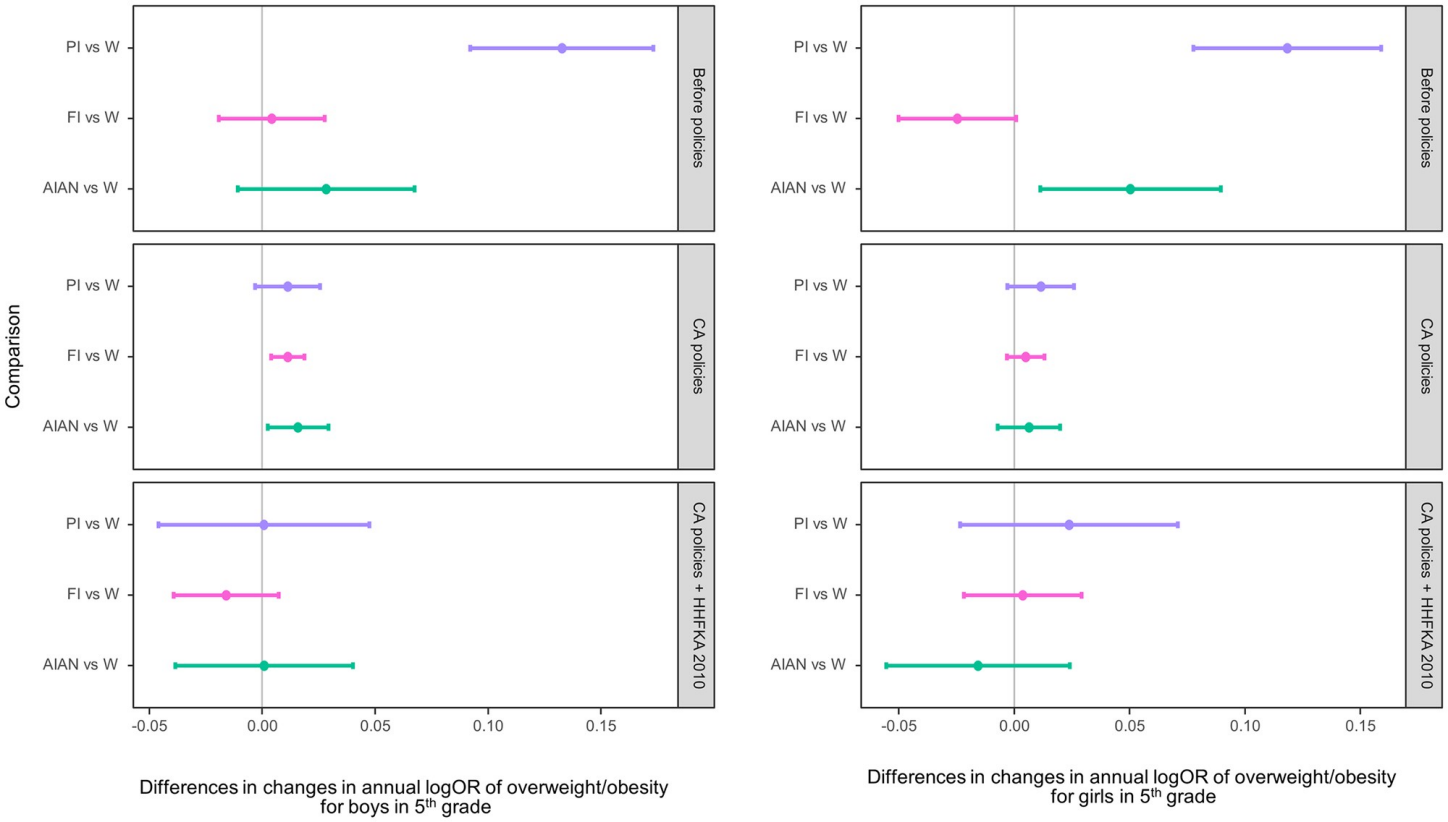
Racial/ethnic differences in adjusted changes in the log odds of overweight/obesity per year comparing the periods 2002–2004 (baseline, no policies in effect), 2005–2012 (after CA policies and before HHFKA 2010), and 2013–2016 (CA policies plus HHFKA 2010) among fifth graders by sex. The dots indicate the point estimates of the yearly changes in the log odds, and the whiskers show the 95% confidence intervals. AIAN, American Indian/Alaska Native; CA, California; FI, children of Filipino origin; HHFKA 2010, Healthy, Hunger-Free Kids Act of 2010; OR, odds ratio; PI, children of Pacific Islander origin; W, White.

**Fig 5:**
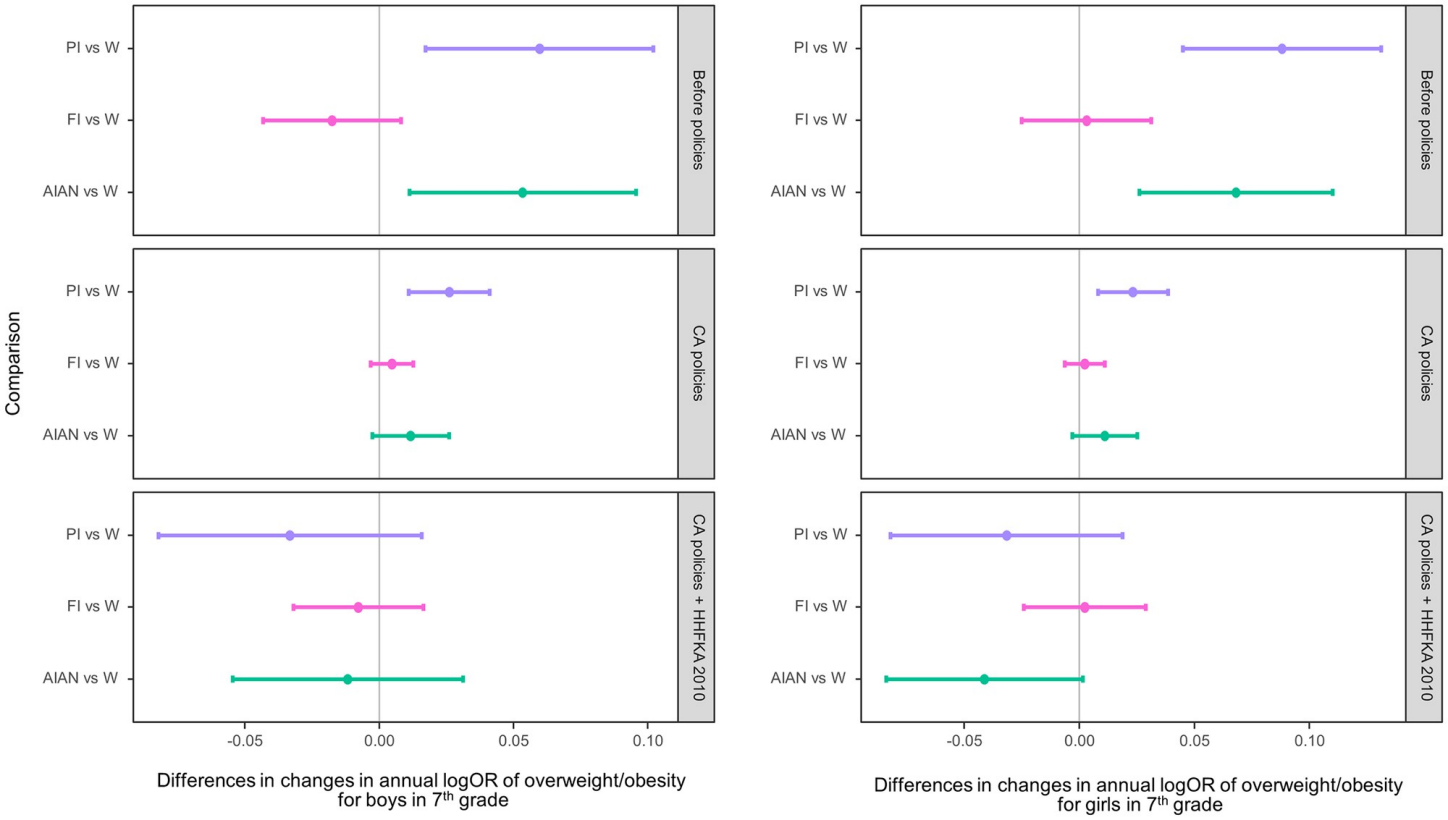
Racial/ethnic differences in adjusted changes in the log odds of overweight/obesity per year comparing the periods 2002–2004 (baseline, no policies in effect), 2005–2012 (after CA policies and before HHFKA 2010), and 2013–2016 (CA policies plus HHFKA 2010) among seventh graders by sex. The dots indicate the point estimates of the yearly changes in the log odds, and the whiskers show the 95% confidence intervals. AIAN, American Indian/Alaska Native; CA, California; FI, children of Filipino origin; HHFKA 2010, Healthy, Hunger-Free Kids Act of 2010; OR, odds ratio; PI, children of Pacific Islander origin; W, White.

Widening disparities of overweight/obesity prevalence were evident between PI and AIAN students and their White peers in both grades during the baseline period (Figs 4 and 5), except for AIAN boys in fifth grade. After the CA CF&B policy took place, overweight/obesity disparities widened for AIAN and FI boys in fifth grade and PI students in seventh grade. When the federal school meals policy took place, there was no clear evidence of increases in overweight/obesity disparities between any of the subgroups and their White peers.

## Discussion

To our knowledge, this is the first study to examine associations of the state and federal school nutrition policies with overweight/obesity among PI, AIAN, and FI children in the US and the policy associations with racial/ethnic obesity disparities. The current study provides evidence to support favorable associations of the school nutrition policies with overweight/obesity prevalence trends among these subgroups and their White peers. However, substantial racial/ethnic differences existed in both prevalence trends and the magnitudes of the association between nutrition policy and overweight/obesity. Large disparities in overweight/obesity prevalence remain between PI and AIAN students and their White peers.

In line with previous research using data from other racial/ethnic populations [12], our study findings suggest that the CA regulations—which were primarily for CF&B—were associated with favorable changes in prevalence trends of overweight/obesity among PI and AIAN students. Of particular importance is that the magnitudes of association were largest for PI students, an encouraging finding given that these students had some of the highest prevalence of overweight/obesity in the FitnessGram population we examined. In addition, while the overweight/obesity prevalence was similarly low for FI and White girls in comparison to other subgroups, we saw stronger evidence of a declining prevalence trend among White girls in fifth grade.

The extent to which the favorable changes in obesity prevalence can solely be attributed to the policies alone remains unclear. However, prior research has observed that availability of CF&B diminished after CF&B policies were implemented [29]. CF&B presence is linked with purchasing and consumption of these products [30], and their removal from school was found to be associated with a decrease in CF&B purchasing in schools, without an increase in consumption of these items at home [31]. These behavioral changes may have played an important role in curtailing the growing childhood obesity epidemic in CA. On the other hand, our study did not find strong and consistent evidence of further reduction in overweight/obesity prevalence across all groups with the addition of the HHFKA 2010, which took effect beginning in 2013. White girls and FI boys in fifth grade experienced additional declines in the prevalence rates, while declines were not clearly observed for the other racial/ethnic subgroups nor among seventh graders. While the HHFKA 2010 policy introduced higher nutritional standards for school meals (e.g., lower saturated fat, *trans*-fat, sodium, and increased fruit and vegetables), CA had banned *trans*-fat and fried foods in school meals starting in 2007, which may partially explain the weaker evidence on HHFKA 2010’s association with childhood obesity for most of the aforementioned subgroups. For students who consume more of CF&B items than on school meals, CF&B policies may be more impactful. The apparent weaker evidence of the HHFKA 2010 policy among the seventh grade students may be explained by higher availability and consumption of CF&B in middle schools compared to elementary schools [32] or by the availability of CF&B outside of schools during the lunch hours. A study, using national data of children 10 to 17 years old, found no overall association between HHFKA2010 and obesity; however, among children in poverty, the odds of obesity declined by 9% each year after the policy was implemented, whereas prior to it, obesity was increasing annually [33]. Additional studies are needed to investigate if these policies have differential effects on obesity specifically among students from understudied racial/ethnic subgroups.

The varying magnitudes of associations across subgroups studied here provide nuanced perspectives on the favorable outcomes of the CA nutrition policies. Differences in sociocultural, environmental, and economic characteristics among these racial/ethnic subgroups may distinctly influence choices of CF&B purchase and consumption. Peers and social norms are thought to play important roles in shaping dietary habits among youth [34–40]. Social norms among peers about snacks, food, and beverage purchasing behaviors in school, near schools, and at home may partially explain the varying magnitudes of association observed in this study. Future studies should assess the effects of food environments within and outside of schools and at home/with families among racial/ethnic populations included in this study. The impact of food environments near schools on weight status also varies among racial/ethnic subgroups [42]. Research has observed greater increases in unhealthy food outlets near Hispanic, African American, and Asian schools located in socioeconomically disadvantaged neighborhoods [41]. However, effects among PI, AIAN, and FI subgroups remain unknown. PI and AIAN subgroups in this study were more likely to attend schools located in less socioeconomically advantaged neighborhoods than White students, which may contribute to greater exposure to unhealthy food outlets in their school neighborhoods.

The finding that disparities between the racial/ethnic subgroups and White students were no longer widening after the nutrition policies is encouraging. Yet, despite these encouraging results, overweight/obesity disparities persisted over the study period. A previous US national study showed that between 2004 and 2007, AI and PI children had 3.9 times higher odds of obesity than Asian children [43]. The current study also saw large gaps between PI and AIAN students—but not for FI students—and their White peers in recent years. These findings highlight the need to disaggregate data whenever possible, to help identify where additional efforts are needed to reduce health disparities across a wider range of population subgroups.

### Strengths and limitations

Our study used an ITS design, one of the strongest study designs for the analyses of repeated cross-sectional data. CA is one of the most populous states in the US with high racial/ethnic diversity, which allowed us to conduct population-level analyses of understudied racial/ethnic groups. The study limitations include the lack of comparison groups who were unexposed to the school nutrition policies as the policies went into effect in the same academic years for all public schools. While the models suggest favorable associations (i.e., smaller increases, flattening, or even decreases in overweight/obesity prevalence), it is not possible to conclude that the changes in trends were directly attributable to the policies we examined. On the other hand, the sample sizes were large and contained much of the student body of these racial/ethnic subgroups within the public school system in the entire state, minimizing the possibility of local variations driving these changes. There were also no competing school nutrition policies that may have affected childhood obesity at the population level. However, the analyses of the effectiveness of these policies among the subgroups included in this study need to be replicated in other contexts to ascertain the generalizability of our findings. Compliance to these policies could not be ascertained. Previous research has shown good compliance to HHFKA 2010, with the vast majority of states being close to 100% compliant [44]. In CA, compliance with CF&B policies was 67% for foods and 78% for beverages in 2008 [45]. We could not account for student-level socioeconomic factors as they were not available in the FitnessGram data set, although we controlled for school-level percentages of students eligible for FRPM as well as school neighborhood income and education levels. Previous studies have reported wide variations in prevalence rates within AIAN across regions and tribes [46,47]. However, we could not analyze differences by location or tribes within PI or AIANs as the information was either only available for some years (PI) or not available at all (AIAN). More studies with subgroup analyses are needed as determinants of health and behaviors vary widely even within these racial/ethnic population subgroups. A more refined understanding of issues facing each racial/ethnic subgroup is a key step to developing effective strategies, particularly in efforts to reduce obesity disparities.

### Implications for future policies and research

The findings from this study have implications for nutrition policies and for future research. While the school nutrition policies we studied sought to make substantial changes to the nutrition standards of foods and beverages served in schools, evidence from this study suggests that such policies need to be strengthened to fully recover from the nation’s childhood obesity epidemic. In addition to improving the nutrient content, school nutrition policies may be augmented with measures to start developing the mindsets and lifelong habits of healthy eating and lifestyles during childhood. For instance, school nutrition programs in Japan and France include education components on healthy eating, local food production, and food cultures, in addition to consideration to nutrient content [48]. Additionally, look-alike products sold outside of the school settings remain as a concern because they are often unhealthy products [49] that look like the healthier version available in schools. Additional nutrition education as well as environmental interventions to offer healthier versions of snacks outside of schools should be considered by policy makers and school officials.

While the racial/ethnic subgroups included in this study constitute relatively small portions of the US population as a whole, they are some of the fastest growing subgroups. The number of people with FI origin in the US quadrupled between 1980 and 2016 [19]. Between 2000 and 2010, the population with PI background grew by 35%, the second fastest growth nationally [50]. Given the high prevalence of youth overweight/obesity among the racial/ethnic subgroups included in this study, our finding on the persisting disparities points strongly to the need to develop targeted interventions to prevent and reduce childhood obesity for these populations.

There is generally sparse evidence in medical research for PI, AIAN, and FI children, partially because historically, smaller racial/ethnic groups have been bundled into one category (i.e., “Other”) in health data [51–55]. National estimates about the burden of diseases in the US often aggregate PI and Asian subgroups, which may result in masked differences in disease risk among specific subgroups. Disaggregated analyses have shown higher burden among PI than Asian subgroups, where the odds of high BMI, hypertension, and diabetes were 1.5 to 2.3 times greater among PI than other Asian subgroups [56]. To more efficiently tackle the childhood obesity epidemic in the US, it is crucial to understand the varying effectiveness of state and federal policies and interventions among different population subgroups, especially those who experienced highest prevalence and disadvantages. Furthermore, these subpopulations may be subject to unique vulnerabilities due to nativity status, cultural influences, and acculturation on diet. Previous research has shown varying degrees of obesity prevalence among youth with immigrant or US-born parents within each racial/ethnic groups [57]. Further research is needed to understand how nativity and acculturation may contribute to differential effects of school nutrition policies among specific subgroups.

The findings from the current study may be useful in efforts to consider strategies to prevent or mitigate epidemics of obesity in other countries undergoing nutrition transition. School nutrition policies may be effective in reducing childhood obesity prevalence in places where the epidemics already exist. They could also serve as preventative measures in places where childhood obesity is not yet common. As we develop strategies tailored toward each racial/ethnic subgroup’s needs in the US, it is also important to share our findings and learn from policies and interventions introduced in other countries around the world.

## Conclusions

This study showed that school nutrition policies were associated with favorable changes in overweight/obesity prevalence trends among PI, AIAN, and FI schoolchildren in CA. However, the prevalence of overweight/obesity remains high among PI and AIAN children and FI boys, and there are wide racial/ethnic obesity disparities between these groups and their White peers. Future subgroup analyses are needed to support the development of additional interventions to reduce childhood obesity among understudied subgroups experiencing disproportionately high burden of obesity.

## Supporting information

S1 Checklist STROBE checklist. STROBE, Strengthening the Reporting of Observational Studies in Epidemiology.
(PDF)

S1 Method Detailed description of the statistical analyses. (PDF)

S1 Table Model estimated values for the yearly changes in log ORs of being overweight obesity by sex, race/ethnicity, and the policy period among fifth graders. CI, confidence interval; OR, odds ratio.
(PDF)

S2 Table Model estimated values for the yearly changes in log ORs of being overweight obesity by sex, race/ethnicity, and the policy period among seventh graders. CI, confidence interval; OR, odds ratio.
(PDF)

S3 Table Comparison of the slopes between 2002–2004 (before policies), 2005–2012 (CA policies only), and 2005–2013 (cumulative comparison) by sex and race/ethnicity among fifth graders. CA, California; CI, confidence interval; OR, odds ratio.
(PDF)

S4 Table Comparison of the slopes between 2002–2004 (before policies), 2005–2012 (CA policies only), and 2005–2013 (cumulative comparison) by sex and race/ethnicity among seventh graders. CA, California; CI, confidence interval; OR, odds ratio.
(PDF)

## Abbreviations

AIAN: American Indian/Alaska Native
BMI: body mass index
CA: California
CDE: California Department of Education
CF&B: competitive foods and beverages
FI: Filipino
FRPM: free or reduced price meals
HHFKA 2010: Healthy, Hunger-Free Kids Act of 2010
ITS: interrupted time series
PACER: Progressive Aerobic Cardiovascular Endurance Run
PI: Pacific Islander
STROBE: Strengthening the Reporting of Observational Studies in Epidemiology
