# Supplementary material for: California and federal school nutrition policies and obesity among children of Pacific Islander, American Indian/Alaska Native, and Filipino origins: Interrupted time series analysis
Source: PLoS Med. 2021 May 24;18(5):e1003596. doi: 10.1371/journal.pmed.1003596 (PMC8143391; doi:10.1371/journal.pmed.1003596)
Supplement: S1 Checklist — STROBE, Strengthening the Reporting of Observational Studies in Epidemiology. (PDF) [file pmed.1003596.s001.pdf]

STROBE Statement

Checklist of items that should be included in reports of observational studies

Section/Topic	Item No	Recommendation	Section	Paragraph number
**Title and abstract**	1	(*a*) Indicate the study's design with a commonly used term in the title or the abstract	Title<br>Abstract	Title<br>Abstract
		(*b*) Provide in the abstract an informative and balanced summary of what was done and what was found	Abstract	Abstract
**Introduction**				
Background/rationale	2	Explain the scientific background and rationale for the investigation being reported	Introduction	1-3
Objectives	3	State specific objectives, including any prespecified hypotheses	Introduction	3
**Methods**				
Study design	4	Present key elements of study design early in the paper	Title<br>Introduction<br>Methods	 3<br>2-12
Setting	5	Describe the setting, locations, and relevant dates, including periods of recruitment, exposure, follow-up, and data collection	Methods	2-10
Participants	6	(*a*) *Cohort study*—Give the eligibility criteria, and the sources and methods of selection of participants. Describe methods of follow-up<br>*Case-control study*—Give the eligibility criteria, and the sources and methods of case ascertainment and control selection. Give the rationale for the choice of cases and controls<br>*Cross-sectional study*—Give the eligibility criteria, and the sources and methods of selection of participants	Methods<br>Results	3,4<br>1
		(*b*) *Cohort study*—For matched studies, give matching criteria and number of exposed and unexposed<br>*Case-control study*—For matched studies, give matching criteria and the number of controls per case	NA	NA
Variables	7	Clearly define all outcomes, exposures, predictors, potential confounders, and effect modifiers. Give diagnostic criteria, if applicable	Methods	5-9, 12-14
Data sources/measurement	8*	For each variable of interest, give sources of data and details of methods of assessment (measurement). Describe comparability of assessment methods if there is more than one group	Methods	5-9
Bias	9	Describe any efforts to address potential sources of bias	Methods	16
Study size	10	Explain how the study size was arrived at	Results	1

Quantitative variables	11	Explain how quantitative variables were handled in the analyses. If applicable, describe which groupings were chosen and why	Methods	2-15
Statistical methods	12	(*a*) Describe all statistical methods, including those used to control for confounding	Methods	11-15
		(*b*) Describe any methods used to examine subgroups and interactions	Methods	12-15
		(*c*) Explain how missing data were addressed	Methods	7, 16
		(*d*) *Cohort study*—If applicable, explain how loss to follow-up was addressed<br>*Case-control study*—If applicable, explain how matching of cases and controls was addressed<br>*Cross-sectional study*—If applicable, describe analytical methods taking account of sampling strategy	NA	NA
		(*e*) Describe any sensitivity analyses	NA	NA

Section/Topic	Item No	Recommendation	Section	Paragraph number
**Results**				
Participants	13*	(a) Report numbers of individuals at each stage of study—eg numbers potentially eligible, examined for eligibility, confirmed eligible, included in the study, completing follow-up, and analysed	Results	1
		(b) Give reasons for non-participation at each stage	NA	Not reported
		(c) Consider use of a flow diagram	NA	Not reported
Descriptive data	14*	(a) Give characteristics of study participants (eg demographic, clinical, social) and information on exposures and potential confounders		
		(b) Indicate number of participants with missing data for each variable of interest		
		(c) *Cohort study*—Summarise follow-up time (eg, average and total amount)	NA	NA
Outcome data	15*	*Cohort study*—Report numbers of outcome events or summary measures over time	NA	NA
		Case-control study—Report numbers in each exposure category, or summary measures of exposure	NA	NA
		Cross-sectional study—Report numbers of outcome events or summary measures	Results	2, Table 1
Main results	16	(*a*) Give unadjusted estimates and, if applicable, confounder-adjusted estimates and their precision (eg, 95% confidence interval). Make clear which confounders were adjusted for and why they were included	Supplemental Materials	S3-S6
		(*b*) Report category boundaries when continuous variables were categorized	Methods	6
		(*c*) If relevant, consider translating estimates of relative risk into absolute risk for a meaningful time period	NA	NA
Other analyses	17	Report other analyses done—eg analyses of subgroups and interactions, and sensitivity analyses	Results	5-10
**Discussion**				
Key results	18	Summarise key results with reference to study objectives	Discussion	1

Limitations	19	Discuss limitations of the study, taking into account sources of potential bias or imprecision. Discuss both direction and magnitude of any potential bias	Methods<br>Discussion	16<br>6
Interpretation	20	Give a cautious overall interpretation of results considering objectives, limitations, multiplicity of analyses, results from similar studies, and other relevant evidence	Discussion	2-10
Generalisability	21	Discuss the generalisability (external validity) of the study results	Discussion	10
**Other Information**				
Funding	22	Give the source of funding and the role of the funders for the present study and, if applicable, for the original study on which the present article is based	Title Page	Funding source

**Give information separately for cases and controls in case-control studies and, if applicable, for exposed and unexposed groups in cohort and cross-sectional studies.*

Note: An Explanation and Elaboration article discusses each checklist item and gives methodological background and published examples of transparent reporting. The STROBE checklist is best used in conjunction with this article (freely available on the Web sites of PLoS Medicine at http://www.plosmedicine.org/, Annals of Internal Medicine at http://www.annals.org/, and Epidemiology at http://www.epidem.com/). Information on the STROBE Initiative is available at www.strobe-statement.org.
