# Supplementary material for: California and federal school nutrition policies and obesity among children of Pacific Islander, American Indian/Alaska Native, and Filipino origins: Interrupted time series analysis
Source: PLoS Med. 2021 May 24;18(5):e1003596. doi: 10.1371/journal.pmed.1003596 (PMC8143391; doi:10.1371/journal.pmed.1003596)
Supplement: S1 Method — (PDF) [file pmed.1003596.s002.pdf]

# Supplemetal Material S2: Modeling details

Multi-level logistic regression models are used to model the trend in the log-odds of overweight/obesity (OVOB) across the study period. Models were estimated separately for males and females, and for 5$^{\text{th}}$ and 7$^{\text{th}}$ grade (4 adjusted models). Specifically, let $Y_{csdt}$ denote OVOB status for child $c$ attending school $s$ in school district $d$ with data collected in the spring of year $t$ (i.e., academic year $(t-1)$ to $t$). Also, define the predictor variables: $Years.Since.Spring2002_t = (t - 2002)$, which represents the years since the beginning of the study period, and $CA.Policy_t = (t - 2005) * I(t \geq 2005)$ and $Fed.Policy_t(t - 2013) * I(t \geq 2013)$ represent linear spline terms with knots placed on the academic years when the policy was implemented (e.g., implementation of the California policy in July of 2004 aligns with data collected in spring of academic year 2004-2005). Given these predictors, the intercept in the model represents the log-odds of OVOB in the spring of 2002, the $Years.Since.Spring2002_t$ captures the slope of the OVOB trend prior to any policies being in place, and the coefficients for the spline terms capture the change in the slope of the OVOB trends following each of the policies. While some policy evaluations allow for a "jump" in the *level* of the outcome when a policy is implemented (French and Heagerty 2008), in this case a jump would imply that children's OVOB levels change immediately upon policy implementation, a biological implausibility given that body weight changes gradually over time. Hence, these terms in the model assume OVOB prevalence follow a continuous, piece-wise linear pattern with inflection points (changes in slope) at the times of policy implementation (see also the supplementary figure with the predicted trend lines estimated from the model).

To simplify the exposition of the model, collect the intercept term and these three predictor variables into the matrix $\boldsymbol{T}$. Finally, let $RE_{r,csdt} = I(RaceEthnicity_{csdt} = r)$ represent an indicator of whether or not the child is of Filipino, American Indian/Native Alaskan, or Pacific Islander origin; White children are the reference group. The multilevel model estimated was:

$$logit[(P(Y_{csdt} = 1)] = \boldsymbol{X_{csdt}}\boldsymbol{\beta_x} + \boldsymbol{T}\boldsymbol{\beta}_w + \sum_{r \in minority} RE_{r,csdt}\boldsymbol{T}\boldsymbol{\beta}_r + \boldsymbol{T}\boldsymbol{b}_d + \boldsymbol{T}\boldsymbol{b}_{sd}.$$

The term $\boldsymbol{X_{csdt}}\boldsymbol{\beta_x}$ represents the adjustment for covariates described in the main text. The parameter $\boldsymbol{\beta}_w$ represents the intercept and the coefficient for the time-related terms among white children, whereas the parameters $\boldsymbol{\beta}_r$ represent the differences in the coefficients comparing children of Filipino, American Indian/Native Alaskan, or Pacific Islander origin relative to white children.

Beyond the fixed effects, i.e., $\boldsymbol{\beta}$'s, the model includes random intercepts and slopes for the trend and spline terms to account for correlation among children's OVOB status within schools and within districts, namely $\boldsymbol{b}_{sd}$ and $\boldsymbol{b}_d$, respectively. These coefficients are modeled as having multivariate normal distributions, namely, $\mathbf{b}_{sd} \sim MVN(\mathbf{b}_d, \boldsymbol{\Sigma})$ and $\mathbf{b}_d \sim MVN(\boldsymbol{\beta}, \boldsymbol{\Gamma})$. The matrices $\boldsymbol{\Sigma}$ and $\boldsymbol{\Gamma}$ are unstructured covariance matrices. While model selection tools are sometimes employed to select the covariance structure for observations nested within larger units, we opted for random effects with unstructured covariances given that: (1) In contrast to other structures for the correlation among observations within schools (e.g., autoregressive), the random effects structure offers the interpretation that the changes in trends associated with the policies could vary between schools and between districts. This is also a recommended approach when data are longitudinal (e.g., French and Heagerty 2008), in our case repeated measures of OVOB within school. (2) The flexibility of the correlation structure among the observations that is induced through an unstructured covariance matrix for the random effects reduces the potential for model misspecification. (3) The large sample size minimizes concerns about loss of degrees of freedom due to a large number of covariance parameters. In sum, this pre-specified covariance structure is one of the most flexible and has a natural interpretation.

The models were estimated using penalized quasi-likelihood, with the `glmmPQL` function in `R`. This approach is versatile in that it is less prone to convergence issues and accommodates the large size of the dataset. To manage the size of the data and shorten computation time, the child-level data were grouped within cells defined by child's age in years, race/ethnicity, and fitness status, within each school (and sex and grade for the four models fitted). This strategy results in enhanced computation because, for example, the data in a school with 500 $5^{\text{th}}$ grade female students can be encapsulated into at most 36 rows of data (3 age groups $\times$ 4 race/ethnicity groups $\times$ 3 fitness statuses) for that school, each row representing `n.subj` children in the age-race/ethnicity/fitness group. Among these `n.subj` children, the number of overweight/obese children is denoted as `x.overweight.obese` $\leq$ `n.subj`. This strategy does not loose any information and results in exactly the same inference compared to using a data set with one row per child. This strategy is possible because all the child-level predictors can be treated as categorical. An example code for 5th grade females is:

```
m.fem.5 = glmmPQL(cbind(x.overweight.obese, n.subj-x.overweight.obese) ~
   YearsSinceSpringYear2002 + CA.Policy + Fed.Policy +
   RaceEthText + RaceEthText:YearsSinceSpringYear2002 +
   RaceEthText:CA.Policy + RaceEthText:Fed.Policy + ...,
   random =  YearsSinceSpringYear2002.d100 + CA.Policy.d100 +
   Fed.Policy.d100|cd.code/cdscode, data = d.fem5, family = binomial,
   control=list(msMaxIter=10000, msMaxEval=10000, niterEM= 1000))
```

In the `random` statement, the predictors were divided by 100 to enhance numerical stability: it is well known that re-scaling enhances numerical stability by re-scaling the magnitude of the variance of the random effects without changing the final inferences.

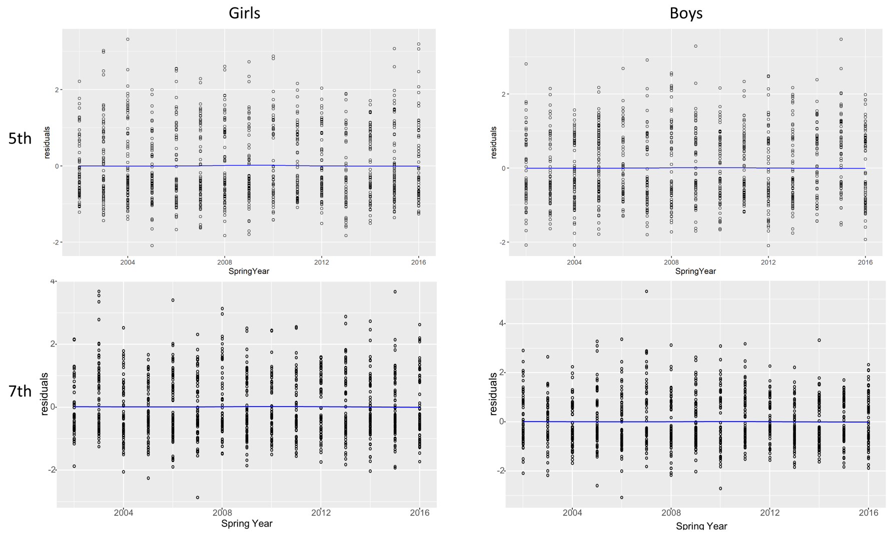


Figure 1: Pearson residuals plotted against calendar time indicate no deviation from the assumed piece-wise linear trend in overweight/obesity prevalence

We used residual diagnostics to check the assumption that OVOB prevalence follows a piece-wise linear trend with pre-specified locations for the knots. We plotted Pearson residuals against calendar year and added a smooth curve to elucidate any deviations from zero among the residuals. The figure (next page) shows no deviations from the assumed piece-wise linear pattern were found 5th grade children. The figure for 7th grade children is similar also demonstrating no deviations (not shown).
