# Supplementary material for: California and federal school nutrition policies and obesity among children of Pacific Islander, American Indian/Alaska Native, and Filipino origins: Interrupted time series analysis
Source: PLoS Med. 2021 May 24;18(5):e1003596. doi: 10.1371/journal.pmed.1003596 (PMC8143391; doi:10.1371/journal.pmed.1003596)
Supplement: S1 Table — CI, confidence interval; OR, odds ratio. (PDF) [file pmed.1003596.s003.pdf]

	Girls in 5th grade			
Before policies (2002-2004)	Unadjusted logOR(95%CI)	p-value	Adjusted logOR (95%CI)	p-value
White	0.027(0.019 to 0.034)	<0.001	0.03(0.02 to 0.041)	<0.001
PI	0.151(0.11 to 0.191)	<0.001	0.149(0.108 to 0.189)	<0.001
AIAN	0.07(0.031 to 0.109)	<0.001	0.081(0.042 to 0.12)	<0.001
FI	0.01(-0.014 to 0.034)	0.423	0.006(-0.02 to 0.031)	0.666
California policies only (2005-2012)				
White	-0.008(-0.012 to -0.005)	<0.001	-0.001(-0.005 to 0.002)	0.474
PI	0.001(-0.013 to 0.016)	0.851	0.01(-0.005 to 0.025)	0.178
AIAN	0.000(-0.014 to 0.013)	0.992	0.005(-0.009 to 0.018)	0.479
FI	-0.005(-0.013 to 0.003)	0.24	0.003(-0.005 to 0.012)	0.417
California and federal policies (2013-2016)				
White	-0.026(-0.035 to -0.016)	<0.001	-0.022(-0.035 to -0.01)	0.001
PI	0.015(-0.032 to 0.063)	0.528	-0.01(-0.066 to 0.046)	0.724
AIAN	-0.05(-0.09 to -0.01)	0.014	-0.044(-0.093 to 0.004)	0.073
FI	-0.029(-0.053 to -0.004)	0.024	-0.024(-0.054 to 0.007)	0.125
	Boys in 5th grade			
Before policies (2002-2004)	Unadjusted logOR(95%CI)	p-value	Adjusted logOR (95%CI)	p-value
White	0.027(0.019 to 0.035)	<0.001	0.011(0.002 to 0.021)	0.024
PI	0.164(0.123 to 0.205)	<0.001	0.144(0.104 to 0.185)	<0.001
AIAN	0.047(0.008 to 0.086)	0.019	0.04(0.001 to 0.079)	0.046
FI	0.034(0.011 to 0.057)	0.003	0.016(-0.008 to 0.039)	0.194
California policies only (2005-2012)				
White	-0.015(-0.018 to -0.012)	<0.001	-0.009(-0.013 to -0.005)	<0.001
PI	-0.009(-0.023 to 0.006)	0.254	0.002(-0.013 to 0.016)	0.808
AIAN	0.005(-0.009 to 0.018)	0.513	0.006(-0.007 to 0.02)	0.353
FI	-0.004(-0.011 to 0.003)	0.304	0.002(-0.006 to 0.01)	0.629
California and federal policies (2013-2016)				
White	-0.02(-0.029 to -0.011)	<0.001	-0.011(-0.023 to 0.002)	0.095
PI	-0.008(-0.055 to 0.04)	0.756	-0.021(-0.077 to 0.035)	0.456
AIAN	-0.033(-0.073 to 0.006)	0.098	-0.026(-0.074 to 0.023)	0.296
FI	-0.042(-0.064 to -0.019)	<0.001	-0.038(-0.066 to -0.01)	0.007
