# Supplementary material for: California and federal school nutrition policies and obesity among children of Pacific Islander, American Indian/Alaska Native, and Filipino origins: Interrupted time series analysis
Source: PLoS Med. 2021 May 24;18(5):e1003596. doi: 10.1371/journal.pmed.1003596 (PMC8143391; doi:10.1371/journal.pmed.1003596)
Supplement: S2 Table — CI, confidence interval; OR, odds ratio. (PDF) [file pmed.1003596.s004.pdf]

	Girls in 7th grade			
Before policies (2002-2004)	Unadjusted logOR(95%CI)	p-value	Adjusted LogOR (95%CI)	p-value
White	0.021(0.012 to 0.031)	<0.001	0.027(0.016 to 0.038)	<0.001
PI	0.113(0.067 to 0.159)	<0.001	0.115(0.072 to 0.158)	<0.001
AIAN	0.094(0.049 to 0.139)	<0.001	0.095(0.053 to 0.137)	<0.001
FI	0.026(-0.003 to 0.055)	0.081	0.03(0.002 to 0.058)	0.035
California policies only (2005-2012)				
White	0.000(-0.003 to 0.004)	0.891	0.008(0.004 to 0.012)	<0.001
PI	0.024(0.008 to 0.041)	0.004	0.031(0.016 to 0.046)	<0.001
AIAN	0.012(-0.003 to 0.028)	0.114	0.019(0.005 to 0.033)	0.010
FI	0.000(-0.009 to 0.009)	0.984	0.01(0.001 to 0.019)	0.024
California and federal policies (2013-2016)				
White	0.004(-0.005 to 0.013)	0.390	0.007(-0.004 to 0.018)	0.197
PI	-0.017(-0.071 to 0.037)	0.538	-0.025(-0.075 to 0.025)	0.336
AIAN	-0.043(-0.088 to 0.003)	0.065	-0.034(-0.077 to 0.008)	0.113
FI	0.006(-0.021 to 0.033)	0.665	0.009(-0.016 to 0.035)	0.476
	Boys in 7th grade			
Before policies (2002-2004)	Unadjusted logOR(95%CI)	p-value	Adjusted LogOR (95%CI)	p-value
White	0.015(0.006 to 0.023)	0.001	0.011(0.001 to 0.021)	0.035
PI	0.109(0.063 to 0.155)	<0.001	0.071(0.028 to 0.113)	0.001
AIAN	0.068(0.022 to 0.114)	0.004	0.064(0.022 to 0.106)	0.003
FI	0.004(-0.022 to 0.03)	0.770	-0.006(-0.032 to 0.019)	0.617
California policies only (2005-2012)				
White	-0.011(-0.014 to -0.008)	<0.001	-0.005(-0.009 to -0.001)	0.025
PI	0.01(-0.006 to 0.027)	0.222	0.021(0.006 to 0.037)	0.005
AIAN	0.004(-0.012 to 0.02)	0.609	0.007(-0.007 to 0.021)	0.337
FI	-0.009(-0.018 to -0.001)	0.025	0.000(-0.008 to 0.008)	0.971
California and federal policies (2013-2016)				
White	0.007(-0.002 to 0.016)	0.109	-0.001(-0.011 to 0.01)	0.923
PI	-0.024(-0.078 to 0.03)	0.388	-0.034(-0.083 to 0.015)	0.174
AIAN	-0.01(-0.057 to 0.036)	0.656	-0.012(-0.055 to 0.03)	0.572
FI	0.004(-0.021 to 0.028)	0.781	-0.008(-0.032 to 0.015)	0.488
