# Supplementary material for: California and federal school nutrition policies and obesity among children of Pacific Islander, American Indian/Alaska Native, and Filipino origins: Interrupted time series analysis
Source: PLoS Med. 2021 May 24;18(5):e1003596. doi: 10.1371/journal.pmed.1003596 (PMC8143391; doi:10.1371/journal.pmed.1003596)
Supplement: S3 Table — CA, California; CI, confidence interval; OR, odds ratio. (PDF) [file pmed.1003596.s005.pdf]

	Girls in 5th grade			
Comparison between 2002-2004 and 2005-2012	Unadjusted logOR(95%CI)	p-value	Adjusted logOR (95%CI)	p-value
White	-0.035(-0.044 to -0.026)	<0.001	-0.032(-0.044 to -0.019)	<0.001
PI	-0.149(-0.199 to -0.099)	<0.001	-0.139(-0.189 to -0.088)	<0.001
AIAN	-0.07(-0.118 to -0.023)	0.004	-0.076(-0.124 to -0.028)	0.002
FI		0.320	-0.002(-0.033 to 0.028)	0.888
Comparison between 2005-2012 and 2013-2016	-0.015(-0.043 to 0.014)			
White	-0.017(-0.028 to -0.006)	0.002	-0.022(-0.035 to -0.01)	0.001
PI	0.014(-0.043 to 0.071)	0.630	-0.01(-0.066 to 0.046)	0.724
AIAN	-0.05(-0.099 to -0.001)	0.046	-0.044(-0.093 to 0.004)	0.073
FI	-0.024(-0.053 to 0.006)	0.114	-0.024(-0.054 to 0.007)	0.125
Comparison between 2002-2004 and 2013-2016				
White	-0.052(-0.064 to -0.04)	<0.001	-0.054(-0.068 to -0.04)	<0.001
PI	-0.135(-0.192 to -0.078)	<0.001	-0.149(-0.206 to -0.092)	<0.001
AIAN	-0.12(-0.171 to -0.07)	<0.001	-0.12(-0.171 to -0.07)	<0.001
FI	-0.038(-0.071 to -0.006)	0.019	-0.026(-0.059 to 0.007)	0.123
	Boys in 5th grade			
Comparison between 2002-2004 and 2005-2012	Unadjusted logOR(95%CI)	p-value	Adjusted logOR (95%CI)	p-value
White	-0.042(-0.052 to -0.033)	<0.001	-0.021(-0.033 to -0.009)	0.001
PI	-0.173(-0.223 to -0.122)	<0.001	-0.142(-0.193 to -0.092)	<0.001
AIAN	-0.042(-0.09 to 0.006)	0.085	-0.033(-0.081 to 0.015)	0.173
FI	-0.038(-0.065 to -0.011)	0.006	-0.014(-0.042 to 0.014)	0.341
Comparison between 2005-2012 and 2013-2016				
White	-0.005(-0.016 to 0.005)	0.332	-0.011(-0.023 to 0.002)	0.095
PI	0.001(-0.056 to 0.058)	0.972	-0.021(-0.077 to 0.035)	0.456
AIAN	-0.038(-0.086 to 0.011)	0.126	-0.026(-0.074 to 0.023)	0.296
FI	-0.038(-0.065 to -0.011)	0.006	-0.038(-0.066 to -0.01)	0.007
Comparison between 2002-2004 and 2013-2016				
White	-0.047(-0.058 to -0.037)	<0.001	-0.032(-0.044 to -0.019)	<0.001
PI	-0.172(-0.228 to -0.115)	<0.001	-0.164(-0.22 to -0.107)	<0.001
AIAN	-0.08(-0.13 to -0.03)	0.002	-0.059(-0.109 to -0.009)	0.021
FI	-0.076(-0.105 to -0.047)	<0.001	-0.052(-0.081 to -0.022)	0.001
