# Supplementary material for: California and federal school nutrition policies and obesity among children of Pacific Islander, American Indian/Alaska Native, and Filipino origins: Interrupted time series analysis
Source: PLoS Med. 2021 May 24;18(5):e1003596. doi: 10.1371/journal.pmed.1003596 (PMC8143391; doi:10.1371/journal.pmed.1003596)
Supplement: S4 Table — CA, California; CI, confidence interval; OR, odds ratio. (PDF) [file pmed.1003596.s006.pdf]

	Girls in 7th grade			
Comparison between 2002-2004 and 2005-2012	Unadjusted logOR(95%CI)	p-value	Adjusted logOR (95%CI)	p-value
White	-0.021(-0.033 to -0.01)	<0.001	-0.019(-0.032 to -0.006)	0.005
PI	-0.089(-0.146 to -0.031)	0.002	-0.084(-0.137 to -0.031)	0.002
AIAN	-0.082(-0.137 to -0.026)	0.004	-0.076(-0.127 to -0.025)	0.003
FI	-0.026(-0.061 to 0.009)	0.14	-0.02(-0.053 to 0.013)	0.237
Comparison between 2005-2012 and 2013-2016				
White	0.004(-0.007 to 0.015)	0.502	-0.001(-0.014 to 0.012)	0.904
PI	-0.041(-0.106 to 0.023)	0.21	-0.056(-0.115 to 0.004)	0.067
AIAN	-0.055(-0.111 to 0)	0.052	-0.053(-0.105 to -0.001)	0.044
FI	0.006(-0.027 to 0.039)	0.717	-0.001(-0.032 to 0.03)	0.963
Comparison between 2002-2004 and 2013-2016				
White	-0.017(-0.029 to -0.005)	0.005	-0.02(-0.035 to -0.005)	0.01
PI	-0.13(-0.195 to -0.065)	<0.001	-0.14(-0.2 to -0.079)	<0.001
AIAN	-0.137(-0.195 to -0.079)	<0.001	-0.129(-0.183 to -0.075)	<0.001
FI	-0.02(-0.056 to 0.016)	0.28	-0.021(-0.056 to 0.015)	0.254
	Boys in 7th grade			
Comparison between 2002-2004 and 2005-2012	Unadjusted logOR(95%CI)	p-value	Adjusted logOR (95%CI)	p-value
White	-0.025(-0.036 to -0.015)	<0.001	-0.016(-0.028 to -0.003)	0.013
PI	-0.099(-0.156 to -0.042)	0.001	-0.049(-0.102 to 0.003)	0.065
AIAN	-0.064(-0.12 to -0.007)	0.027	-0.057(-0.109 to -0.006)	0.029
FI	-0.013(-0.045 to 0.018)	0.407	0.007(-0.024 to 0.037)	0.667
Comparison between 2005-2012 and 2013-2016				
White	0.018(0.007 to 0.029)	0.001	0.004(-0.009 to 0.017)	0.528
PI	-0.034(-0.099 to 0.03)	0.3	-0.055(-0.113 to 0.003)	0.063
AIAN	-0.015(-0.071 to 0.042)	0.614	-0.019(-0.071 to 0.033)	0.467
FI	0.013(-0.017 to 0.043)	0.397	-0.009(-0.037 to 0.02)	0.56
Comparison between 2002-2004 and 2013-2016				
White	-0.007(-0.019 to 0.004)	0.203	-0.012(-0.026 to 0.002)	0.1
PI	-0.133(-0.198 to -0.068)	<0.001	-0.105(-0.164 to -0.046)	0.001
AIAN	-0.078(-0.137 to -0.019)	0.009	-0.077(-0.131 to -0.022)	0.006
FI	0.000(-0.033 to 0.033)	0.98	-0.002(-0.034 to 0.03)	0.907
